# Tumor-infiltrating monocytes/macrophages promote tumor invasion and migration by upregulating S100A8 and S100A9 expression in cancer cells

**DOI:** 10.1038/onc.2016.107

**Published:** 2016-04-18

**Authors:** S Y Lim, A E Yuzhalin, A N Gordon-Weeks, R J Muschel

**Affiliations:** 1 CRUK/MRC Oxford Institute for Radiation Oncology, University of Oxford, Oxford, UK

## Abstract

Myeloid cells promote the development of distant metastases, but little is known about the molecular mechanisms underlying this process. Here we have begun to uncover the effects of myeloid cells on cancer cells in a mouse model of liver metastasis. Monocytes/macrophages, but not granulocytes, isolated from experimental liver metastases stimulated migration and invasion of MC38 colon and Lewis lung carcinoma cells. In response to conditioned media from tumor-infiltrating monocytes/macrophages, cancer cells upregulated *S100a8* and *S100a9* messenger RNA expression through an extracellular signal-related kinase-dependent mechanism. Suppression of S100A8 and S100A9 in cancer cells using short hairpin RNA significantly diminished migration and invasion in culture. Downregulation of S100A8 and S100A9 had no effect on subcutaneous tumor growth. However, colony size was greatly reduced in liver metastases with decreased invasion into adjacent tissue. In tissue culture and in the liver colonies derived from cancer cells with knockdown of S100A8 and S100A9, MMP2 and MMP9 expression was decreased, consistent with the reduction in migration and invasion. Our findings demonstrate that monocytes/macrophages in the metastatic liver microenvironment induce S100A8 and S100A9 in cancer cells, and that these proteins are essential for tumor cell migration and invasion. S100A8 and S100A9, however, are not responsible for stimulation of proliferation. This study implicates S100A8 and S100A9 as important mediators of tumor cell aggressiveness, and highlights the therapeutic potential of S100A8 and S100A9 for interference of metastasis.

## Introduction

Myeloid cells populate the tumor microenvironment. These myeloid cells are highly heterogeneous with cells of both the monocytic and granulocytic lineages, and have considerable phenotypic plasticity with both positive and negative effects on tumor growth and metastasis.^1, 2^ The balance between anti-tumor and pro-tumor functions can be dependent on polarization state, interaction with the tumor microenvironment and/or the tumor type.^3, 4, 5^ Understanding the actions of myeloid cells on cancer cells could be essential in distinguishing, and possibly manipulating, the positive from the negative effectors.^6, 7^

Distant metastasis remains the main cause of cancer-related death. During the early stages of metastasis, tumor cells acquire migratory and invasive characteristics, allowing movement into surrounding extracellular matrix and tissues, intravasation into blood vessels, and dissemination via the circulation. Following extravasation into target tissues, tumor cells initiate metastatic colonization, in part by evading tumor surveillance and instigating an angiogenic response.^8, 9^ Myeloid cells have been shown to affect all of these steps.

We previously examined the effects of infiltrating myeloid cells on experimental liver metastases generated by intrasplenic inoculation of MC38 colon and Lewis lung carcinoma (LLC) cells. These metastatic colonies were infiltrated by CD11b^+^ cells comprising granulocytes and monocytes/macrophages. Depletion of CD11b^+^ cells led to markedly reduced colony growth. To begin to understand how these effects were mediated, we isolated cancer cells after removal of the CD11b^+^ myeloid cells. Angiopoietin-like 7 (ANGPTL7) expression was greatly reduced in the cancer cells. Enforced overexpression of ANGPTL7 inhibited growth of liver metastases and subcutaneous tumors. In the same study, we also found that S100A8 and S100A9 expression in cancer cells was altered by removal of the CD11b^+^ cells.^10^

Here we explored the significance of S100A8 and S100A9 induction by the myeloid cells in the tumor microenvironment. S100A8 and S100A9 are calcium-binding proteins that form homo- and heterocomplexes (S100A8/A9) that are important for their biological activity,^11^ although some functions are independent of heterocomplex formation.^12^ These proteins stimulate chemotaxis, cell migration and adhesion,^13^ but also have anti-inflammatory roles in oxidant scavenging, tissue repair and resolution of inflammation.^14^ The effects of S100A8 and S100A9 are dependent on concentration, post-translational modifications,^15, 16^ oligomeric states and/or the microenvironment.^12^

S100A8 and S100A9 are expressed to a greater extent in colorectal, prostate and breast cancers.^17, 18^ In colorectal cancers, increased S100A8 and S100A9 expression correlated with differentiation, Dukes stage and lymph node metastasis.^19^ Similarly, in prostate cancer, S100A8 and S100A9 were expressed at increased levels in high-grade adenocarcinomas compared with benign tissues.^20^ S100A8 and S100A9 expression in breast cancer correlated with HER2 expression and lymph node metastasis.^21^ These studies indicate that S100A8 and S100A9 levels are elevated in cancer tissues compared with normal and benign tissues, and their increased expression is associated with tumor aggressiveness and metastasis.

In the published literature, S100A8 and S100A9 are reported as predominantly expressed within tumors by immune cells, and their expression can stimulate the recruitment of myeloid^22, 23^ and myeloid-derived suppressor cells^24^ to promote pre-metastatic niche formation, tumor growth and metastasis.^25^ S100A8 and S100A9 are also expressed by tumor cells,^26^ and although there have been many studies detailing the functions of stromal-derived S100A8 and S100A9, little is known about the effects of tumor-derived S100A8 and S100A9. In this study, we report that monocytes/macrophages induce *S100a8* and *S100a9* messenger RNA (mRNA) expression in cancer cells in an extracellular signal-related kinase (ERK)-dependent manner. S100A8 and S100A9 expression in cancer cells was critical for invasion by liver metastases. These findings detail a novel molecular mechanism through which tumor-derived expression of S100A8 and S100A9, regulated by infiltrating monocytes/macrophages, dictates a more aggressive phenotype.

## Results

### Monocytic myeloid cells promote tumor cell proliferation, migration and invasion

**Figure 1:**
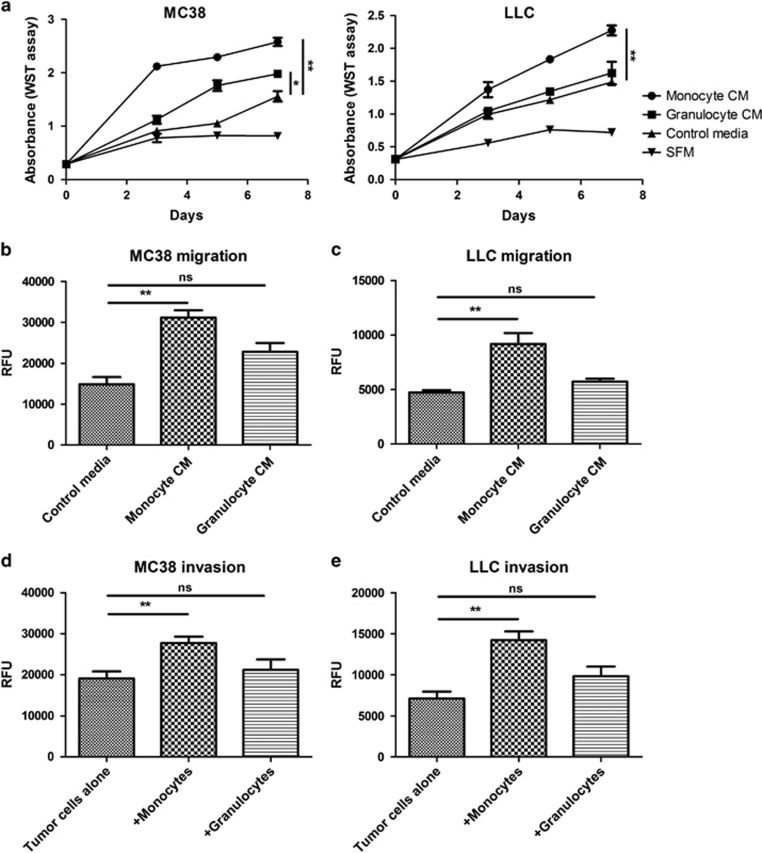
Effects of CD11b^+^ monocytic and granulocytic cells on tumor cell proliferation, migration and invasion. (**a**) MC38 and LLC cells were cultured in conditioned media (CM) derived from monocytes/macrophages or granulocytes, or in control media and their proliferation assessed using the water-soluble tetrazolium (WST) assay. Migration of (**b**) MC38 and (**c**) LLC cells in response to control, monocyte/macrophage- or granulocyte-conditioned media was assessed using the Cytoselect Migration Assay. Invasion of (**d**) MC38 and (**e**) LLC cells co-cultured with monocytes/macrophages or granulocytes was assessed using the Cytoselect Invasion Assay. Three independent experiments were performed, *P<0.05, **P<0.01. NS, not significant; RFU, relative fluorescence unit.

To determine the mechanisms through which myeloid cells support the development of liver metastasis, we first investigated whether augmentation of some tumor cell properties by myeloid cells could be duplicated in tissue culture. In previous work, we found that the tumor colonies generated by MC38 or LLC cancer cells were infiltrated by CD11b^+^ myeloid cells that could be divided into three distinct groups based on their Gr1 expression (Gr1^high^, Gr1^mid^ and Gr1^low^). The Gr1^high^ cells were predominantly granulocytes, whereas the Gr1^mid^ and Gr1^low^ cells were monocytes/macrophages.^27^ The latter two groups were interrelated as Gr1^mid^ cells could give rise to Gr1^low^ cells.^10^ Gr1^high^ granulocytes and Gr1^mid/low^ monocytes/macrophages were isolated from liver metastases by fluorescence-activated cell sorting (FACS), and after 3 days of culture, conditioned media were collected and added to cultures of MC38 and LLC cancer cells. The conditioned medium from tumor-infiltrating monocytes/macrophages significantly increased proliferation of MC38 and LLC cells. Conditioned medium from the granulocytic population increased MC38 cell proliferation to a lesser extent, and had no effect on LLC cell proliferation (Figure 1a). Conditioned medium from monocytes/macrophages also enhanced the migratory capability of MC38 and LLC cells, whereas granulocyte-conditioned medium had little effect (Figures 1b and c). Moreover, co-culture with monocytes/macrophages significantly increased invasion of MC38 and LLC cells through Matrigel. In contrast, co-culture with granulocytes did not enhance invasion (Figures 1d and e). Overall, these results indicate that monocytic myeloid cells infiltrating liver metastases stimulated tumor cell growth, migration and invasion.

### Monocytes/macrophages upregulate S100a8 and S100a9 expression in tumor cells in an ERK-dependent manner

**Figure 2:**
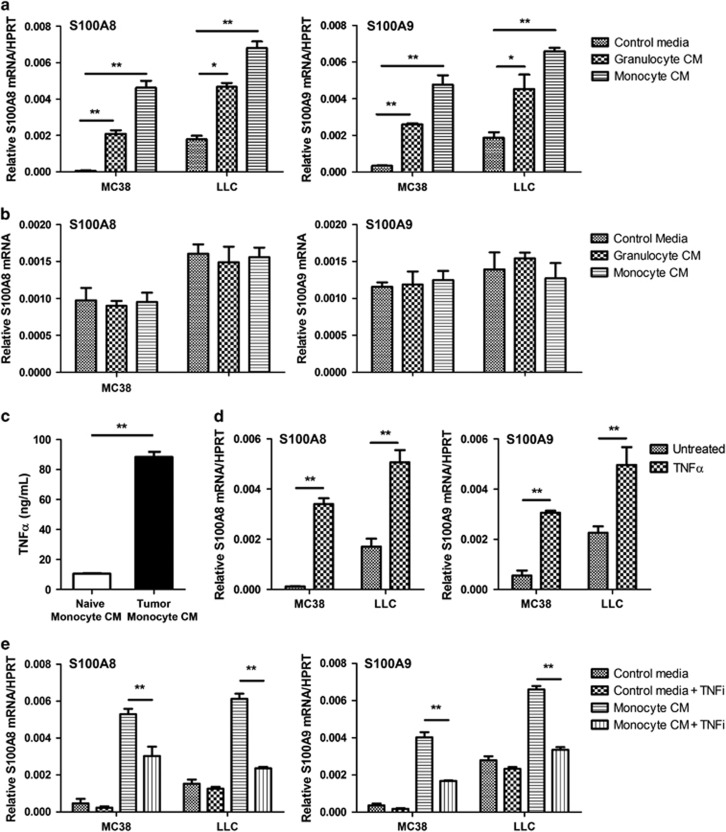
Effects of monocytes/macrophages and granulocytes on S100A8 and S100A9 expression. Expression of (**a**) *S100a8* and *S100a9* mRNA in MC38 and LLC cells was assessed by quantitative PCR (qPCR) after culture in tumor-infiltrating monocyte/macrophage- or granulocyte-conditioned media. Expression of (**b**) *S100a8* and *S100a9* mRNA in MC38 and LLC cells was also assessed after culture in conditioned media derived from naive monocytes/macrophages or granulocytes. (**c**) TNFα expression in conditioned media derived from tumor-infiltrating (tumor monocyte CM) and naive (naive monocyte CM) monocytes/macrophages was assessed by enzyme-linked immunosorbent assay. (**d**) *S100a8* and *S100a9* mRNA in MC38 and LLC cells was assessed by qPCR untreated or after treatment with 100 ng/ml TNFα. (**e**) *S100a8* and *S100a9* mRNA in MC38 and LLC cells was assessed by qPCR after culture in control or tumor-infiltrating monocyte/macrophage-conditioned media with or without anti-TNFα (TNFi) inhibitory antibodies. For all studies, three independent experiments were performed, *P<0.05, **P<0.01 compared with controls.

Culture of MC38 and LLC cancer cells in conditioned media from tumor-infiltrating monocytes/macrophages and granulocytes induced *S100a8* and *S100a9* (Figure 2a) mRNA expression. In contrast, conditioned media from naIve monocytes/macrophages and granulocytes isolated from normal livers did not alter *S100a8* and *S100a9* mRNA expression in the cancer cells (Figure 2b). Culture of human colorectal cancer cells HT29, HCT116 and LOVO in monocytes/macrophage-conditioned media similarly upregulated *S100a8* and *S100a9* mRNA expression in the cancer cells, indicating that induction of these genes by the conditioned media was not restricted to murine cell lines (Supplementary Figure 1).

We found ~10-fold more tumor necrosis factor-α (TNFα) in tumor-infiltrating monocyte/macrophage-conditioned media compared with the conditioned media from naive monocytes/macrophages (Figure 2c), suggesting TNFα as a possible candidate for the induction of S100A8 and S100A9. Recombinant mouse TNFα (100 ng/ml) upregulated both *S100a8* and *S100a9* mRNA in MC38 and LLC cells (Figure 2d). Blockade of TNFα using an inhibitory antibody in the monocyte/macrophage-conditioned media negated *S100a8* and *S100a9* induction in both MC38 and LLC cells (Figure 2e).

**Figure 3:**
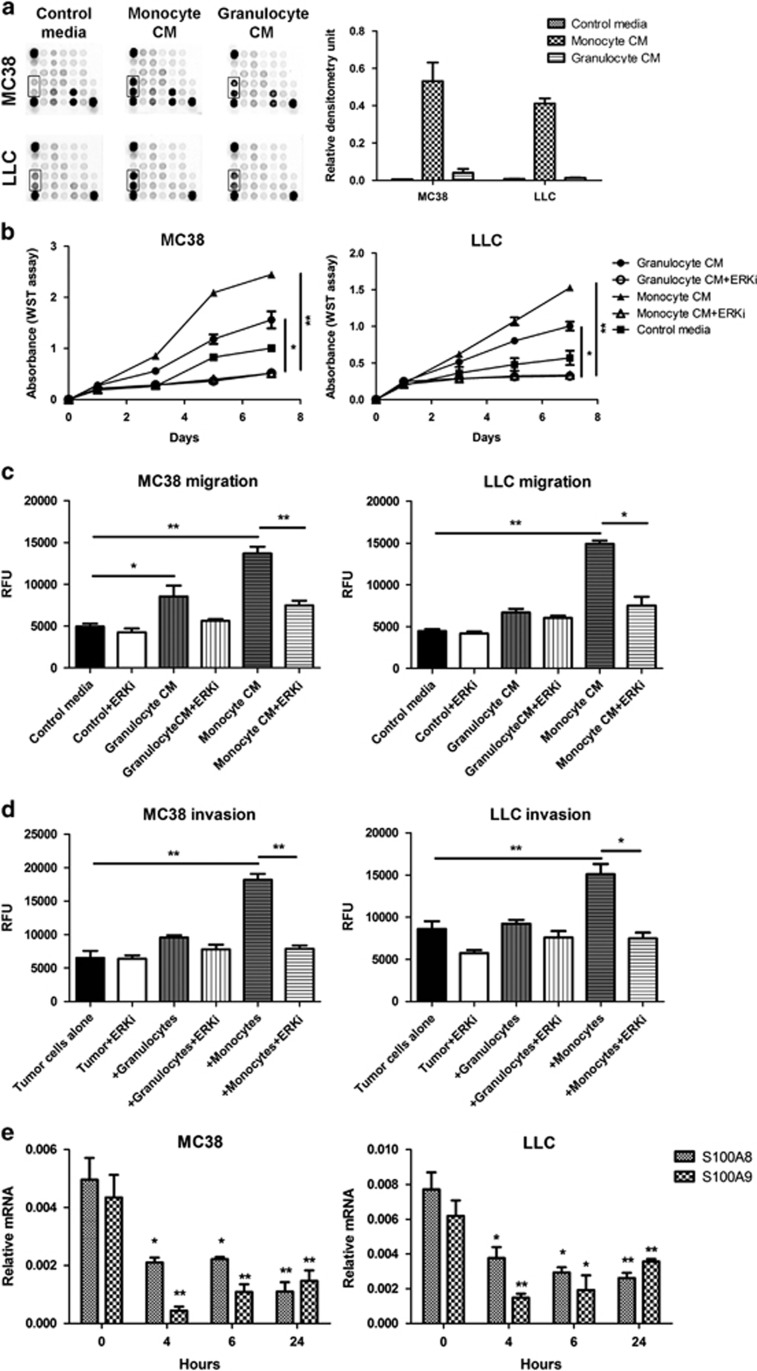
Effects of ERK signaling in MC38 and LLC tumor cells. (**a**) MC38 and LLC cells were cultured in monocyte/macrophage- and granulocyte-conditioned media, and activation of the ERK signaling pathway (boxed area) was assessed using the PathScan Intracellular Signaling Array Kit by measuring densitometry values. Three independent experiments were performed, and representative image is shown. (**b**) Proliferation, (**c**) migration and (**d**) invasion of MC38 and LLC cells in response to monocytes/macrophages or granulocytes were tested in the presence of the ERK inhibitor U0126 (10 μm; +ERKi) using the WST or Cytoselect assays. Three independent experiments were performed, *P<0.05, **P<0.01. (**e**) Expression of *S100a8* and *S100a9* mRNA in MC38 and LLC cells cultured in monocyte/macrophage-conditioned medium was assessed by quantitative PCR after treatment with 10 μm ERK inhibitor U0126 for the indicated times. Three independent experiments were performed, *P<0.05, **P<0.01 compared with time 0 h.

To assess the changes in the cancer cells resulting from exposure to the conditioned media, we examined the signaling pathways using the PathScan Intracellular Signaling Array Kit (Cell Signaling Technology, Leiden, Netherlands). Conditioned medium from monocytes/macrophages provoked a pronounced increase in ERK1/2 phosphorylation (Thr202/Tyr204) compared with controls. Granulocyte-conditioned medium activated ERK1/2, but to a lesser extent. Changes in phosphorylation levels of other signaling pathway indicators on the array were not apparent (Figure 3a).

ERK signaling is an important regulator of cancer cell proliferation, as well as migration and invasion in many settings,^28^ and is activated by TNFα (100 ng/ml) in MC38 and LLC cells (Supplementary Figure 2), and in several other cancer cell lines.^29, 30^ Inhibition of ERK1/2 with 10 μm U0126 (Cell Signaling Technology) in MC38 and LLC cells suppressed tumor cell proliferation in response to monocyte/macrophage- and granulocyte-conditioned media (Figure 3b). ERK inhibition also blocked augmentation of migration and invasion by monocytes/macrophages (Figures 3c and d). Because the levels of S100A8 and S100A9 in untreated MC38 and LLC cells were low, we first induced S100A8 and S100A9 expression by stimulating cancer cells with monocyte/macrophage-conditioned medium. Pharmacological inhibition of ERK signaling abolished the increased expression of *S100a8* and *S100a9* mRNA in MC38 and LLC cells after stimulation with monocyte/macrophage-conditioned medium. In both cancer cell lines, *S100a8* mRNA levels were reduced at 4 h and remained low for 24 h after ERK inhibition. *S100a9* mRNA expression was also reduced after 4 h, but recovered over 24 h albeit not to basal levels (Figure 3e).

Collectively, these data suggest that monocytes/macrophages in liver metastases induced tumor cell proliferation, migration and invasion, and upregulated *S100a8* and *S100a9* expression through TNFα-induced ERK activation. We now asked which of these functions were mediated by S100A8 and S100A9.

### S100A8 and S100A9 mediate increased cancer cell migration and invasion

**Figure 4:**
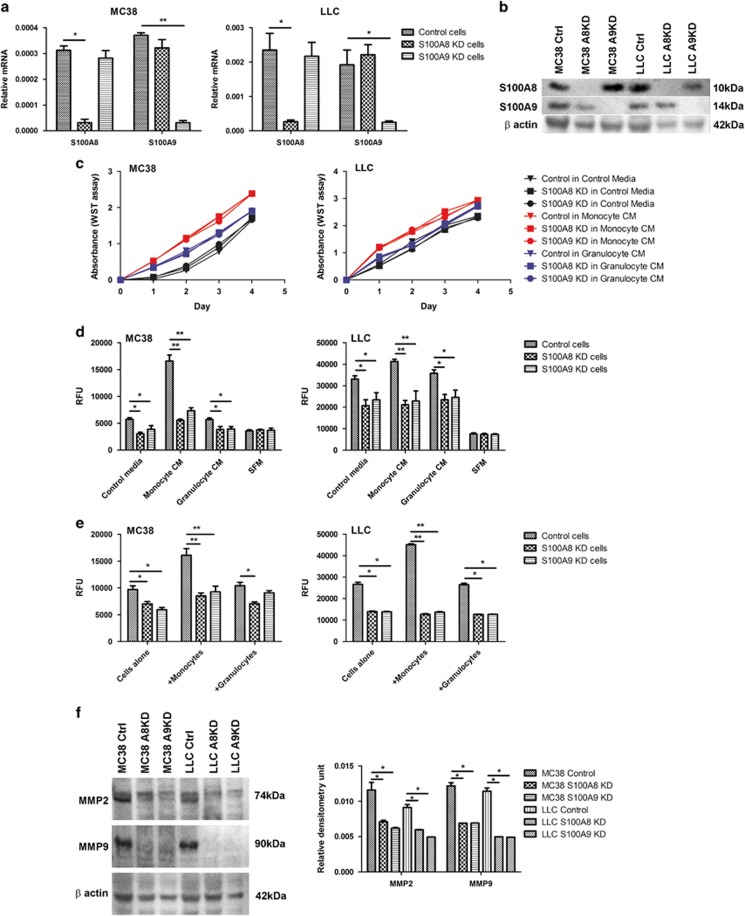
Effects of S100A8 and S100A9 knockdown on tumor cell proliferation, migration and invasion. Expression of S100A8 and S100A9 in MC38 and LLC cells transfected with lentiviral S100A8- (S100A8 KD), S100A9- (S100A9 KD) or scrambled control (control)-shRNA was assessed by (**a**) quantitative PCR and (**b**) western blotting. Three independent experiments were performed, and representative image is shown. (**c**) Proliferation of control, S100A8 KD or S100A9 KD MC38 and LLC cells was assessed using the WST assay. (**d**) Migration and (**e**) invasion of control, S100A8 KD or S100A9 KD MC38 and LLC cells in response to monocytes/macrophages or granulocytes were assessed using the Cytoselect assays. For all assays, three independent experiments were performed, *P<0.05, **P<0.01. (**f**) Expression of MMP2 and MMP9 in control, S100A8 KD or S100A9 KD MC38 and LLC cells was determined by western blotting and semi-quantitated using densitometry analysis. Three independent experiments were performed, and representative image is shown. KD, knockdown.

To determine the effects of S100A8 and S100A9 on tumor cell proliferation, migration and invasion, we downregulated both the genes in MC38 and LLC cells using lentiviral short hairpin RNA (shRNA). Transfection of MC38 and LLC cells with S100A8-shRNA decreased basal *S100a8* mRNA levels by 2- and 2.5-fold, respectively (Figure 4a). S100A9-shRNA transfection reduced basal *S100a9* mRNA levels by 4-fold in MC38 cells and by 2.5-fold in LLC cells (Figure 4a). S100A8 knockdown did not affect *S100a9* mRNA expression or vice versa in either cell line. Decreased S100A8 and S100A9 protein levels in MC38 and LLC cells were confirmed by western blotting (Figure 4b).

Knockdown of S100A8 or S100A9 did not affect MC38 or LLC cell proliferation when they were cultured in control, monocyte/macrophage- or granulocyte-conditioned media (Figure 4c). We had previously shown that myeloid cells suppressed ANGPTL7 expression in cancer cells, leading to decreased tumor cell proliferation,^10^ and in keeping with this, S100A8 or S100A9 knockdown did not alter *ANGPTL7* mRNA levels (Supplementary Figure 3). Despite having no effect on proliferation, S100A8 or S100A9 knockdown significantly decreased the migration of MC38 and LLC cells in response to control medium, or to stimulation with monocyte/macrophage- and granulocyte-conditioned media (Figure 4d). Invasion of MC38 and LLC cells through Matrigel was similarly reduced by reduction of S100A8 or S100A9 expression (Figure 4e). S100A8 and S100A9 form heterocomplexes. Here knockdown of either S100A8 or S100A9 greatly reduced migration and invasion. These results are consistent with the importance of complex formation for biological activity.^11, 13^ Thus, monocytes/macrophages enhanced tumor cell migration and invasion, but not proliferation, through S100A8 and S100A9 upregulation in the cancer cells.

### Knockdown of S100A8 and S100A9 decrease tumor-derived MMP2 and MMP9

Matrix metalloproteinases (MMPs) are known to have key roles in tumor cell migration and invasion.^31^ S100A8 and S100A9 were previously shown to stimulate MMP2 and MMP9 expression in breast and gastric cancer cells.^32, 33^ We found that S100A8 and S100A9 had similar effects in MC38 and LLC cells. S100A8 and S100A9 knockdown significantly decreased MMP2 and MMP9 protein levels in the cancer cells (Figure 4f). Reduction in MMP2 and MMP9 expression after S100A8 and S100A9 knockdown in the cancer cells suggests their regulation by the S100 proteins, and is consistent with decreased tumor cell migration and invasion.

### Knockdown of S100A8 and S100A9 decrease metastatic burden but have little effect on tumor growth

**Figure 5:**
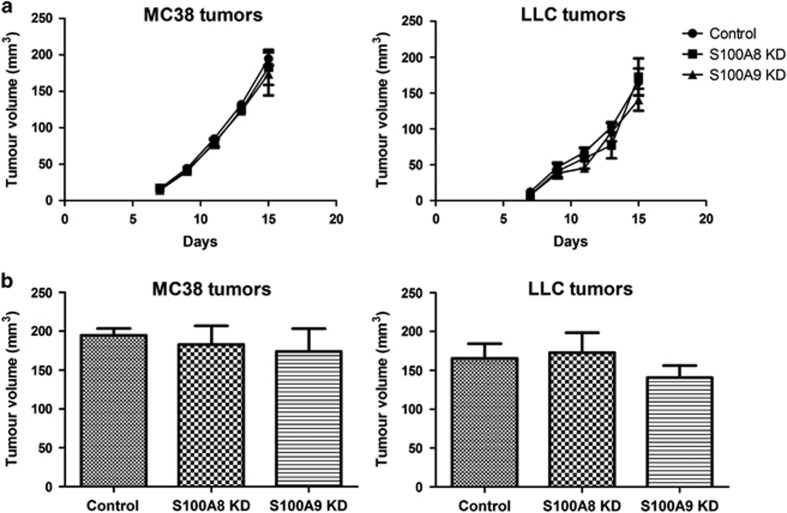
Effects of S100A8 and S100A9 knockdown on MC38 and LLC tumorigenicity. (**a**) Subcutaneous tumor growth in C57BL/6 mice following injection of control, S100A8 KD or S100A9 KD MC38 and LLC cells was monitored using calipers at the indicated times. (**b**) Volumes of subcutaneous tumors from control, S100A8 KD or S100A9 KD MC38 and LLC injected cells were assessed after 2.5 weeks. *n*=6 per group.

**Figure 6:**
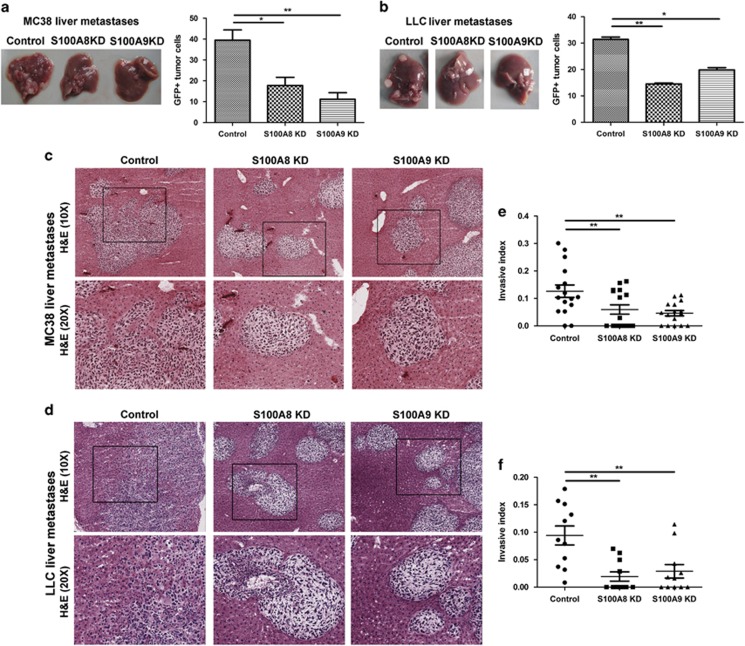
Effects of S100A8 and S100A9 knockdown on liver metastases. Morphological examination of tumor-bearing livers from mice inoculated with control, S100A8 KD or S100A9 KD (**a**) MC38 and (**b**) LLC cells. Metastatic tumor burden in the liver was assessed by the percentage of GFP^+^ tumor cells. Tissue sections from tumor-bearing livers of mice inoculated with control, S100A8 KD or S100A9 KD (**c**) MC38 and (**d**) LLC cells were stained with hematoxylin and eosin (H&E), and representative images taken at × 10 and × 20 magnification (inset of boxed area) are shown. The invasive index of (**e**) MC38 and (**f**) LLC liver metastases was assessed according to 1−(non-invading area/total area) to calculate the ratio of invasive area. Only the tumor colonies of similar size in the three experimental groups were compared. *n*=6–8 per group with two to three tumor colonies imaged per mouse, *P<0.05, **P<0.01. KD, knockdown.

To assess the effects of S100A8 and S100A9 *in vivo*, we injected S100A8-shRNA-, S100A9-shRNA- and scrambled control-shRNA-transfected MC38 and LLC cells subcutaneously into syngeneic mice. Subcutaneous tumor growth was not affected, consistent with the absence of the effects on proliferation in tissue culture (Figures 5a and b). In contrast, liver metastasis burden was significantly altered. S100A8-shRNA-, S100A9-shRNA- or scrambled control-shRNA-transfected MC38 and LLC cells were injected intrasplenically into syngeneic mice. Mice inoculated with S100A8-shRNA- or S100A9-shRNA-transfected MC38 or LLC cells had considerably reduced metastatic tumor burden compared with controls (Figures 6a and b). Despite the reduction in colony numbers and size, staining of tumor-bearing tissue sections revealed little difference in tumor cell proliferation between the knockdown and control groups based on Ki67 as a marker (Supplementary Figure 4A). Apoptosis (Supplementary Figure 4B) and tumor vasculature (Supplementary Figure 4C) were similarly unchanged between the experimental groups.

On examination of the hematoxylin- and eosin-stained liver sections (Figures 6c and d), tumor colonies appeared to be better demarcated, with well-circumscribed borders in the S100A8 and S100A9 knockdown groups compared with controls. Tumor colonies of similar size in the three experimental groups were compared with exclude possible influence of tumor size on invasion. More invasive extensions of the tumor colonies into the adjacent liver were evident in controls compared with the S100A8 or S100A9 knockdown cells. To verify this impression, we compared the area of the invasion front of each individual tumor colony and found the invasive areas to be significantly reduced when S100A8 and S100A9 were downregulated in both MC38 and LLC cells (Figures 6e and f). Comparison of tumor colonizes of different sizes in the three experimental groups also showed markedly reduced invasive areas in the S100A8 and S100A9 knockdown groups (Supplementary Figure 6). Thus, S100A8 and S100A9 mediate invasion by the liver colonies in analogy to their effects in tissue culture.

**Figure 7:**
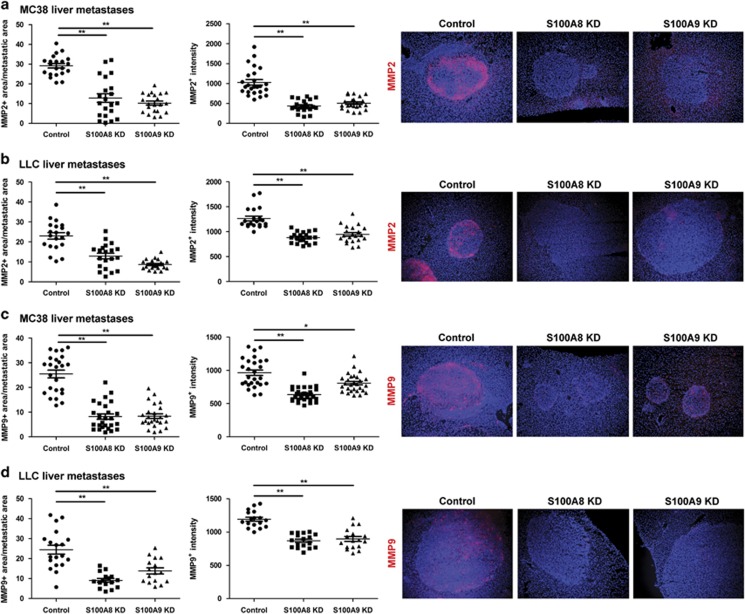
Effects of S100A8 and S100A9 knockdown on MMP2 and MMP9 expression in liver metastases. Tissue sections from tumor-bearing livers of mice inoculated with control, S100A8 KD or S100A9 KD (**a**) MC38 and (**b**) LLC cells were stained for MMP2, and the density and intensity of staining were quantified. Tissue sections of (**c**) MC38 and (**d**) LLC liver metastases were also stained for MMP9, and the density and intensity of staining quantified. Representative images taken at × 10 magnification are shown on the right, *n*=5–6 per group with two to three to three tumor colonies imaged per mouse, *P<0.05, **P<0.01. KD, knockdown.

As S100A8 and S100A9 levels affected MMP2 and MMP9 expression in tissue culture, we examined their expression in the *in vivo* liver colonies. MMP2 and MMP9 expression was localized around the invasive edge of the liver colonies. Staining area and intensity of MMP2 (Figures 7a and b) and MMP9 (Figures 7c and d) in the MC38 and LLC tumor colonies were notably decreased in the S100A8 and S100A9 knockdown groups compared with controls.

## Discussion

In this study, we show that tumor-infiltrating monocytes/macrophages regulate the expression of S100A8 and S100A9 in cancer cells. These proteins enhanced migration and invasion of cancer cells in liver metastases. In the same system, we previously found that monocytes/macrophages suppressed ANGPTL7 expression in cancer cells. This suppression promoted tumor proliferation and angiogenesis.^10^ Expression of *S100a8* and *S100a9* was independent of *ANGPTL7* expression (Supplementary Figure 3). Taken together, these studies suggest that monocytes/macrophages enhance malignancy in liver metastases through two separate mechanisms: (1) stimulating tumor cell migration and invasion through S100A8 and S100A9 induction; and (2) promoting tumor proliferation and angiogenesis by downregulating ANGPTL7.

MC38 and LLC liver colonies are heavily infiltrated by granulocytes and monocytes/macrophages in our liver metastasis model. Monocytes/macrophages from tumor-bearing livers, but not normal livers, expressed high levels of TNFα and stimulated *S100a8* and *S100a9* expression. TNFα and activation of ERK by monocytes/macrophages were essential for *S100a8* and *S100a9* induction. Monocyte/macrophage-conditioned medium stimulated ERK signaling in cancer cells. Granulocytes did not provoke ERK activation to the same extent, consistent with their reduced ability to induce *S100a8* and *S100a9* expression in cancer cells.

Many studies have shown that expression of S100A8 and S100A9 by stromal cells recruits myeloid cells to the tumor microenvironment.^22, 24^ Here we describe a distinct functional role for cancer cell-derived S100A8 and S100A9. S100A8 forms heterocomplexes with S100A9.^34, 35^ Downregulation of either S100A8 or S100A9 in cancer cells reduced tumor migration and invasion. All such actions have been ascribed to the S100A8/A9 heterocomplex. In our experiments, blocking the expression of either the S100A8 or the S100A9 component via shRNA similarly reduced migration and invasion in tissue culture, and liver metastasis formation and liver metastasis invasion *in vivo*. Our observations are thus consistent with the heterocomplex being the active form.^11, 13^

Downregulation of S100A8 and S100A9 was further accompanied by decreased MMP2 and MMP9 expression in cancer cells, both in culture and in metastatic liver colonies. MMPs are often upregulated in tumor cells and promote cancer cell migration and invasion through degradation of the extracellular matrix.^36^ Increased MMP expression has been associated with tumor progression and metastasis, and their inhibition diminished metastatic incidence in several cancer types.^37, 38^ In our model, the reduction in metastatic tumor burden may be caused, in part, by decreased MMP2 and MMP9 expression, leading to diminished capacity of the cancer cells to migrate and invade. This appears to be essential in liver metastases, likely because growth requires invasion into the adjacent tissue due to relative non-compliance of the liver tissue. In contrast, at subcutaneous sites, there is less physical resistance to colony expansion. Moreover, given that metastases can and often do derive from other metastases, this makes the S100A8- and S100A9-mediated invasive capacity of cancer cells more ominous.

The mechanisms mediating S100A8 and S100A9 regulation of MMP2 and MMP9 expression in cancer cells are not yet known but may involve their calcium-binding activity. Expression of MMP2^39^ and MMP9^40^ is dependent on the intracellular calcium levels and in keeping with this, S100A8 and S100A9 could regulate their expression by modulating calcium concentration in cancer cells.^41, 42^ Indeed, we found that stimulation of MC38 and LLC cells with monocyte/macrophage-conditioned media, which induces S100A8 and S100A9 (Figures 2a and b), decreased intracellular calcium levels compared with unstimulated cancer cells. Decrease in calcium levels upon stimulation was abrogated when S100A8 and S100A9 were knocked down in the cancer cells (Supplementary Figure 7). This finding suggests a potential mechanism, and points to functional differences between tumor-derived and stromal-derived S100A8 and S100A9. S100A8 and S100A9 were expressed by stromal cells stimulated myeloid cell recruitment,^24^ but we found no difference in myeloid cell infiltration in liver metastases when S100A8 and S100A9 were downregulated in the cancer cells (Supplementary Figure 8). In our model, tumor-derived S100A8 and S100A9 have distinct effects apart from myeloid cell recruitment, one of which is to promote cancer cell invasion. While we demonstrate the effects mediated intracellularly through alterations in calcium levels, tumor-derived S100A8 and S100A9 are likely to have other effects both intracellular and/or extracellular in the tumor microenvironment, both in our model and in other tumor settings.

Overall, this study supplies a novel mechanism to explain how monocytic myeloid cells stimulate metastatic progression and highlights the multiple effects and mechanisms employed by myeloid cells during liver metastasis. We demonstrate that tumor-infiltrating monocytes/macrophages induced S100A8 and S100A9 expression in cancer cells to promote migration and invasion. These findings support S100A8 and S100A9 as molecular markers of tumor aggressiveness, in keeping with previous studies showing S100A8 and S100A9 overexpression correlating with poor pathological parameters.^19, 21^ Furthermore, our findings suggest that S1008 and S100A9 may serve as useful clinical targets for anti-metastasis therapy. Indeed, inhibition of S100A9 using quinoline-3-carboxamide derivatives such as tasquinimod has shown anti-tumor effects in several pre-clinical models, through modulation of the tumor microenvironment; tasquinimod inhibited myeloid-derived suppressor cell recruitment and infiltration, leading to enhanced tumor immunity and decreased angiogenesis.^43^ Our study further indicates that targeting S100A9 may have additional direct effects on cancer cells, by suppressing their invasive and migratory capabilities.

## Materials and methods

### Cell lines

MC38 and LLC cells stably expressing green fluorescent protein (GFP) were generated and cultured as described.^27^ Human HT29, HCT116 and LOVO colorectal cancer cell lines were purchased from American Type Culture Collection (Manassas, VA, USA) and cultured in Dulbecco's modified Eagle's medium supplemented with 10% fetal bovine serum. For all cell lines, mycoplasma testing using the MycoAlert Mycoplasma Detection kit (Lonza, Slough, UK) was performed every 3 months, and cell line DNA typing (DNA Diagnostics Centre, Fairfield, OH, USA) was performed in October 2014. Cells were regularly assessed based on their morphology before use. Monocytes/macrophages (CD11b^+^/Gr1^mid/low^) and granulocytes (CD11b^+^/Gr1^high^) were isolated from single-cell suspensions of tumor-bearing livers by FACS based on CD11b and Gr1 expression. These cells have been previously characterized based on their morphology, and Ly6C, Ly6G and CD11c expression.^27^ Isolated myeloid cells were cultured in 2% fetal bovine serum in Roswell Park Memorial Institute (RPMI; control media) media for 72 h before conditioned media was collected. MC38 or LLC cells (1 × 10^5^ cells per well) were co-cultured for 8 h in the myeloid cell-conditioned media or with 100 ng/ml recombinant mouse TNFα (Invitrogen, Paisley, UK) in 12-well plates before gene analysis. The ERK inhibitor U0126 (10 μm; Cell Signaling Technology) was added to the cell culture in some instances. To generate stable knockdown of S100A8 and S100A9, MC38 and LLC cells were transfected with lentivirus-mediated shRNA targeting S100A8 or S100A9 or the scrambled shRNA control (MISSION shRNA, Sigma, Poole, UK). Transfected cells were selected with 2.5 μg/ml puromycin.

### Animal models

Animal procedures were carried out in accordance with the UK Animal (Scientific Procedures) Act 1986 and followed local ethics review. Female C57BL/6 mice at 6–8 weeks of age were purchased from Charles River Laboratories (Kent, UK). To develop liver metastasis, tumor cells (5 × 10^5^ in 100 μl phosphate-buffered saline) were injected into the spleen of anesthetized female C57BL/6 mice, the spleen was removed after 1 min and mice were killed 2–3 weeks after tumor cell injection. Subcutaneous tumors formed after injection of tumor cells (5 × 10^5^ in 50 μl phosphate-buffered saline) were injected subcutaneously into the left flank of anesthetized C57BL/6 mice. Volumes were determined by caliper measurement of the length, width and height of each tumor. For all animal work, at least five or more mice were randomized and included in each experimental group, and all animals used were included in the analysis. Animal studies were not blinded during data analysis.

### FACS analysis

Single-cell suspensions were prepared from tumor-bearing livers as previously described.^10^ Cell suspensions were stained in the presence of Mouse BD Fc Block (BD Biosciences, Oxford, UK). Antibodies used in FACS included rat anti-mouse CD11b PE-Cy7 (eBioscience, Hatfield, UK, 25-0112-82), rat anti-mouse Gr1 PE (eBioscience, 12-5931-82) and isotype controls rat IgG2a PE and rat IgG2b PE-Cy7 (eBioscience). Flow cytometry was performed using a FACSCalibur flow cytometer (BD Biosciences) and analyzed using FlowJo software version 7.2.5 (Tree Star, Ashland, OR, USA). Myeloid cells were sorted into granulocyte (CD11b^+^/Gr1^high^) or monocyte/macrophage (CD11b^+^/Gr1^mid/low^) subsets using a MoFlo XDP High-Speed Cell Sorter (Beckman Coulter, High Wycombe, UK) by the antibodies described above.

### Western blotting

Cells were lyzed in radioimmunoprecipitation assay buffer and lysates were assayed by western blot analysis for the expression of S100A8 (Abcam, Cambridge, UK, ab92331), S100A9 (Abcam, ab105472), MMP2 (Abcam, ab92536) and MMP9 (Abcam, ab137867), ERK1/2 (Cell Signaling, #9126S) or phosphorylated ERK1/2 (Cell Signaling, #4695S) using the indicated antibodies.

### Immunohistochemistry and immunofluorescence staining

Mouse livers were snap-frozen, sectioned (10–12 μm) and fixed in acetone, before blocking with serum and incubating with primary antibodies at 4 °C overnight. Primary antibodies included anti-mouse Ki67 (Vector Laboratories, Peterborough, UK, VP-RM04), anti-mouse CD31 (Abcam, ab7388), anti-mouse MMP2 (Abcam, ab92536) and anti-mouse MMP (Abcam, ab137867) or isotype rabbit and rat IgG controls (R&D Systems, Oxford, UK). Fluorescence-conjugated secondary antibodies (Molecular Probes, Invitrogen; A21429 and A21208) were added and tissues were mounted with Vectashield containing 4',6-diamidino-2-phenylindole (Vector Laboratories). Apoptotic cells were stained using the ApopTag Fluorescein In Situ Apoptosis Detection Kit according to the manufacturer's instruction (Millipore, Watford, UK). Images were acquired using an inverted epifluorescence microscope (DM IRBE, Leica Microsystems) with a digital camera (C4742-95; Hamamatsu Photonics, Hertfordshire, UK) or an inverted confocal microscope (LSM-710, Carl Zeiss Microimaging, Hertfordshire, UK) and processed using ImageJ (National Institute of Health, Bethesda, MD, USA), Adobe Photoshop CS4 (Adobe Systems, San Jose, CA, USA) or ImageScope Version 12.1.0.5029 (Aperio Technologies, Oxford, UK). Tissue sections were also fixed in acetone before staining with hematoxylin and eosin according to the standard protocol. Invasion index was determined by the ratio of the invasive area to total tumor area, as calculated as 1−(non-invading area/total area) from hematoxylin- and eosin-stained images as previously described.^44^

### Cell proliferation, migration and invasion assays

Cells were cultured in serum-free RPMI for 24 h before seeding at 1 × 10^3^ cells per well in 96-well plates in control or myeloid cell-conditioned media. Cell proliferation was measured according to the manufacturer's instruction (Roche, Lewes, UK) at the indicated times using the water-soluble tetrazolium (WST)-1 reagent (10 μl added to each well at end point). Cell migration and invasion were assayed using the CytoSelect 96-well Cell Migration and Invasion Assay according to the manufacturer's instruction (Cell Biolabs, Cambridge, UK). In brief, MC38 and LLC cells expressing GFP were resuspended in serum-free RPMI containing 0.1% bovine serum albumin (BSA). For the migration assay, granulocyte- or monocyte/macrophage-conditioned media or RPMI media containing 2% fetal bovine serum (control media) was added to the bottom chamber (100 μl per well). Tumor cell suspensions (5 × 10^5^ cells per ml) in serum-free RPMI containing 0.1% BSA were added to the top insert and cells were incubated at 37 °C for 8 h before assessing the number of tumor cells in the bottom chamber using GFP fluorescence. For the invasion assays, tumor cells (5 × 10^5^ cells per ml), alone or mixed with isolated granulocytes or monocytes/macrophages (1 × 10^5^ cells per ml), were added to the top chamber in serum-free RPMI containing 0.1% BSA, whereas RPMI media containing 2% fetal bovine serum was added to the bottom chamber. Cells were incubated at 37 °C for 8 h before assessing the number of tumor cells in the bottom chamber using GFP fluorescence.

### Intracellular calcium assay

Intracellular calcium levels were measured using the FLUO-4 NW Calcium Assay kit (Molecular Probes, Invitrogen). In brief, 3 × 10^4^ MC38 and LLC control, S100A8 or S100A9 knockdown cells were plated in 96-well plates and incubated with control or monocyte/macrophage-conditioned media for 8 h. Ionomycin (10 μm; Sigma) was added as a positive control. Media was removed and cells incubated with dye loading solution according to the manufacturer's protocol. Plates were read on a fluorescent plate reader at 494/516 nm.

### Intracellular signaling pathway analysis

MC38 or LLC cells were lyzed 24 h after culture in granulocyte- or monocyte/macrophage-conditioned media. Cell lysates were assayed using the PathScan Intracellular Signaling Array Kit according to the manufacturer's protocol (Cell Signaling Technology). Densitometric measurements were made using ImageJ (National Institute of Health). MC38 and LLC cells were cultured alone or with 100 ng/ml recombinant mouse TNFα (Invitrogen), lyzed and assayed for total ERK using MEK1/2 antibodies (Cell Signaling Technology, #9126S) and for phosphorylated ERK using p44/42 MAPK antibodies (Cell Signaling Technology, #4695S).

### TNFα expression analysis and inhibition

Expression of TNFα in conditioned media was assayed using the TNF alpha mouse enzyme-linked immunosorbent assay kit (Abcam). TNFα activity was neutralized in the myeloid cell-conditioned media using 50 μg/ml mouse TNF-α antibody (R&D Systems; AF-410-SP).

### Gene expression analysis

RNA was isolated with Trizol (Invitrogen) according to the manufacturer's instructions, and complementary DNA (cDNA; 0.5 μg) was synthesized using the SuperScript VILO cDNA synthesis kit (Invitrogen) or the Tetro cDNA synthesis kit (Bioline, London, UK). Real-time PCR reaction mixtures were prepared with the Platinum SYBR Green qPCR Supermix-UDG (Invitrogen) and performed on a Stratagene MX3005P thermocycler (Stratagene, Cambridge, UK). Data were analyzed using the ΔCT method and normalized to hypoxanthine–guanine phosphoribosyl transferase (HPRT). Primers used included mouse S100A8 (forward: 5′-CCGTCTTCAAGACATCGTTTGA-3′, reverse: 5′-GTAGAGGGCATGGTGATTTCCT-3′), mouse S100A9 (forward: 5′-GCCAACAAAGCACCTTCTCA-3′, reverse: human S100A8 (forward: 5′-GGGATGACCTGAAGAAATTGCTA-3′, reverse: 5′-TGTTGATATCCAACTCTTTGAACCA-3′), human S100A9 (forward: 5′-GTGCGAAAAGATCTGCAAAATTT-3′, reverse 5′-GGTCCTCCATGATGTGTTCTATGA-3′), mouse HPRT (forward: 5′-GCAGTACAGCCCCAAAATGG, reverse: 5′-AACAAAGTCTGGCCTGTATCCAA-3′) and human HPRT (forward: 5′-ATAAGCCAGACTTTGTTGG-3′, reverse: ATAGGACTCCAGATGTTTCC-3′).

### Statistical analysis

Statistical analyses were performed using GraphPad Prism (version 5, GraphPad Software, San Diego, CA, USA). For all experiments, data were expressed as mean±s.e.m. and analyzed by unpaired two-sided Student's *t*-tests, or one-way analysis of variance with Bonferroni post test for multiple comparisons. A *P*-value of<0.05 was considered significant.
