# Supplementary material for: Tumor-infiltrating monocytes/macrophages promote tumor invasion and migration by upregulating S100A8 and S100A9 expression in cancer cells
Source: Oncogene. 2016 Apr 18;35(44):5735–45. doi: 10.1038/onc.2016.107 (PMC4961254; doi:10.1038/onc.2016.107)
Supplement: Supplementary Figures [file onc2016107x1.pdf]

SUPPLEMENTARY FIGURES

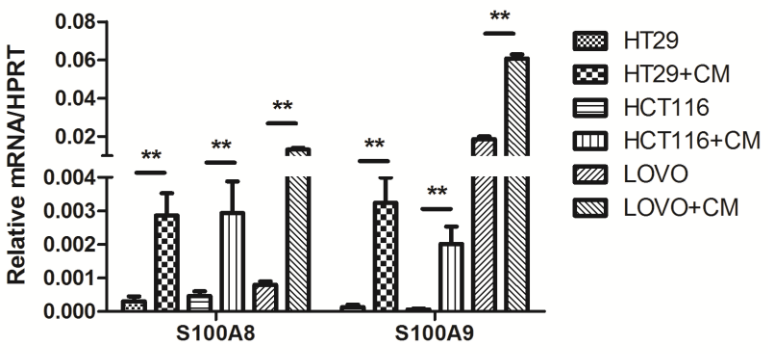

Supplementary Figure 1: S100A8 and S100A9 expression in human colon cancer cells. Expression of *S100a8* and *S100a9* mRNA in human colon cancer cells HT29, HCT116 and LOVO were assessed by qPCR after culture in control or monocyte/macrophage-conditioned media (+CM). Three independent experiments were performed, **p<0.01.

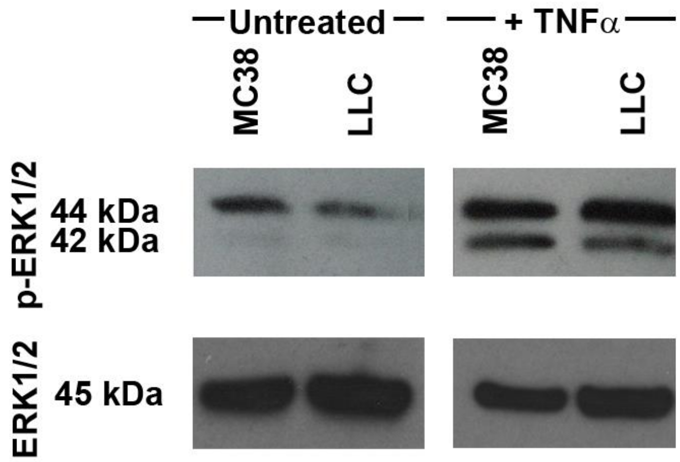

Supplementary Figure 2: ERK activation in MC38 and LLC cells by TNFα. MC38 and LLC cells were culture alone or with 100 ng/mL TNFα. Cells were lyzed and assayed for total ERK and phosphorylated ERK expression by Western blotting.

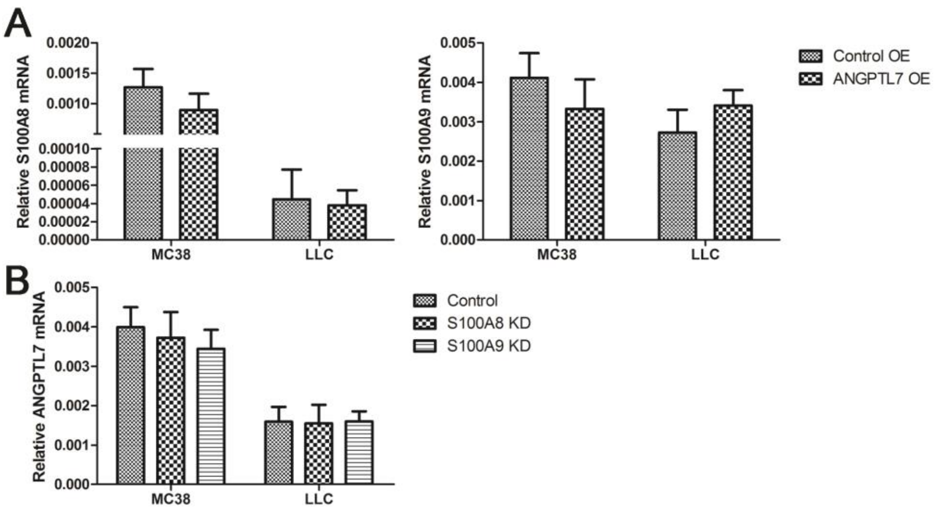

Supplementary Figure 3: Expression of S100A8, S100A9 and ANGPTL7 in MC38 and LLC tumor cells. Expression of *S100a8* and *S100a9* mRNA in control (Control OE) or ANGPTL7 overexpressing (ANGPTL7 OE) MC38 and LLC cells were assessed by qPCR (A). Expression of *ANGPTL7* mRNA in MC38 and LLC cells transfected with lentiviral S100A8- (S100A8 KD), S100A9- (S100A9 KD) or scrambled control- (Control) shRNA were assessed by qPCR (B). Three independent experiments were performed.

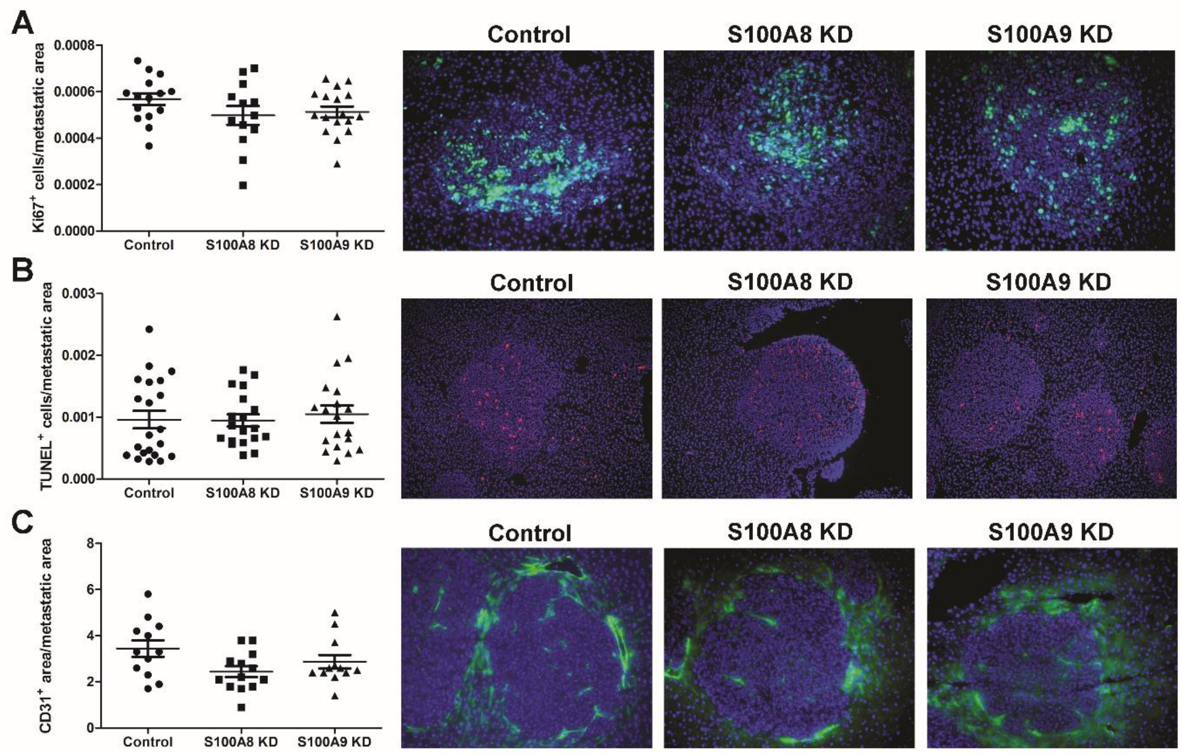

Supplementary Figure 4: Effects of S100A8 and S100A9 knockdown on MC38 tumor proliferation, apoptosis and vasculature. Tissue sections from tumor bearing livers of mice inoculated with control, S100A8 KD or S100A9 KD MC38 cells were stained for Ki67 to detect proliferating cells (A), using TUNEL to detect apoptotic cells (B) and for CD31 to detect tumor vasculature (C). Representative images of staining are shown at 10x magnification on the right, n=6-8 mice/group with 2-3 tumor colonies imaged per mouse.

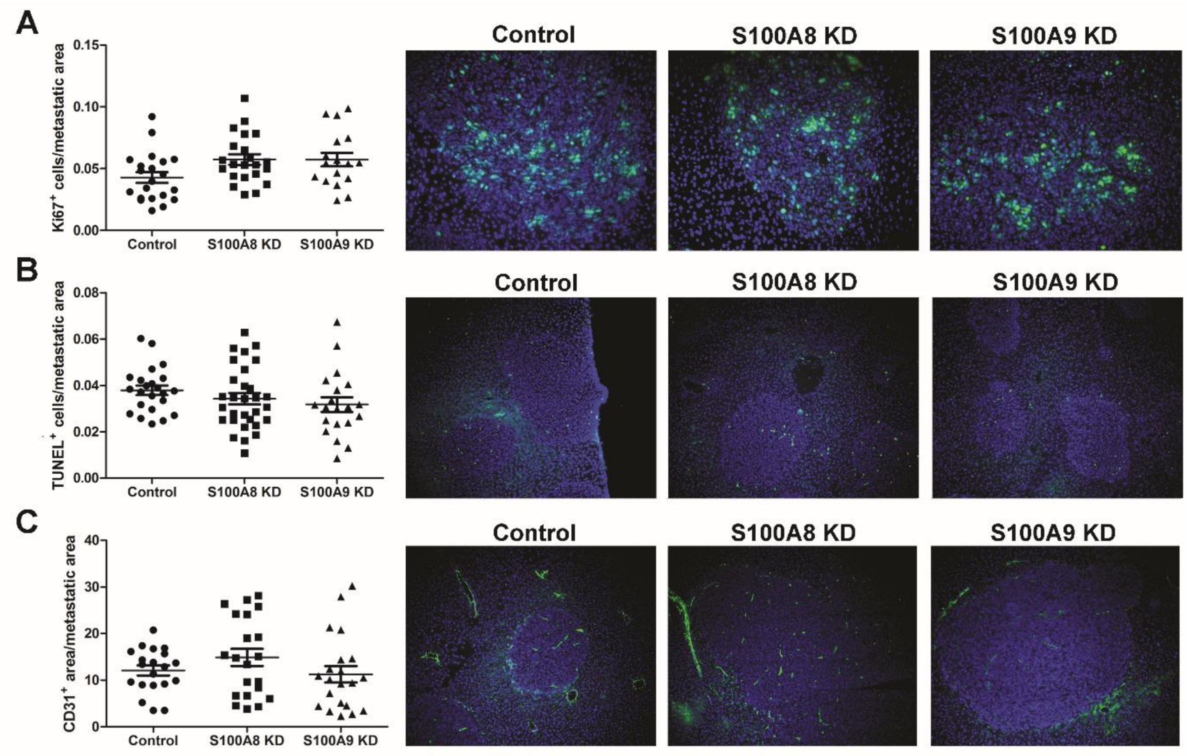

Supplementary Figure 5: Effects of S100A8 and S100A9 knockdown on LLC tumor cell proliferation, apoptosis and vasculature. Tissue sections from tumor bearing livers of mice inoculated with control, S100A8 KD or S100A9 KD LLC cells were stained for Ki67 to detect proliferating cells (A), using TUNEL to detect apoptotic cells (B) and for CD31 to detect tumor vasculature (C). Representative images of staining are shown at 10x magnification on the right, n=5-6 mice/group, with 2-3 tumor colonies imaged per mouse.

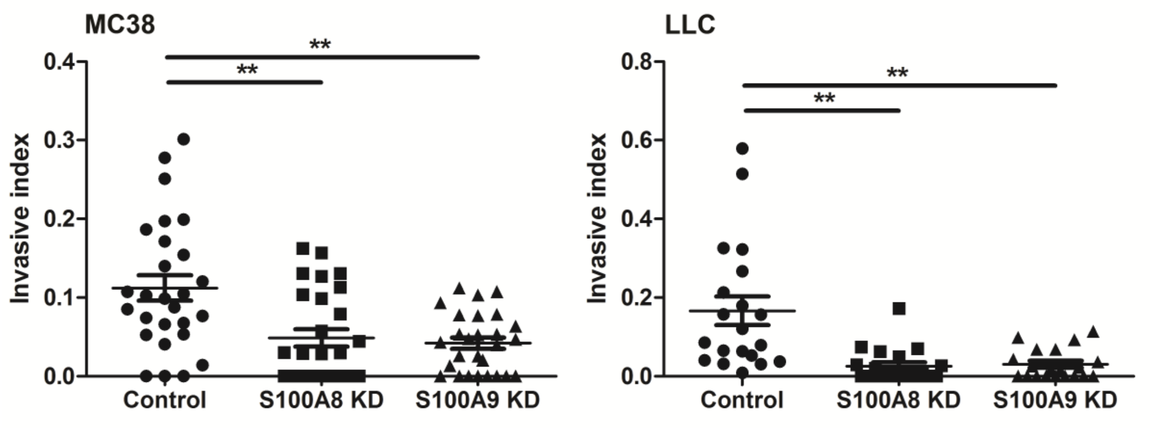

Supplementary Figure 6: Effects of S100A8 and S100A9 knockdown on tumor invasion in liver metastasis. The invasive index of MC38 and LLC liver metastases was assessed according to 1-(non-invading area/total area) to calculate ratio of invasive area in tumor colonies of all sizes. n=6-8/group with 2-3 tumor colonies imaged per mouse, *p<0.05, **p<0.01.

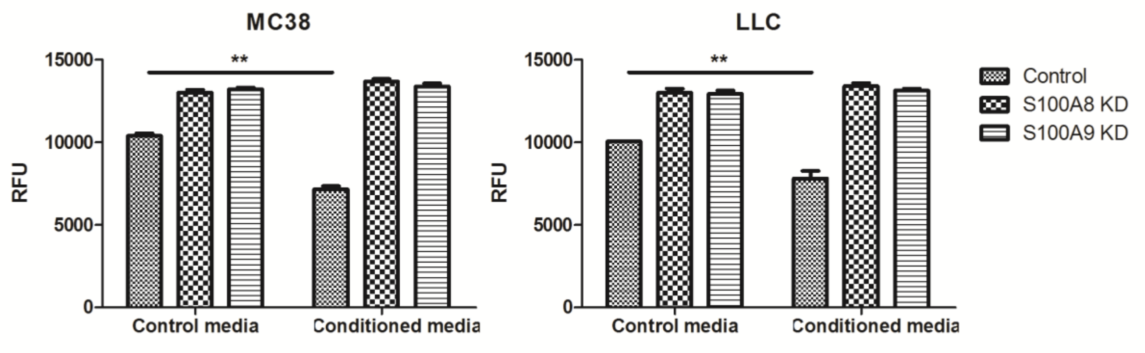

Supplementary Figure 7: Effects of S100A8 and S100A9 expression on intracellular calcium levels. Control, S100A8 or S100A9 knockdown MC38 and LLC cells were stimulated with control or monocyte/macrophage-conditioned media for 8 h before measuring intracellular calcium levels. Three independent experiments were performed, *p<0.05, **p<0.01.

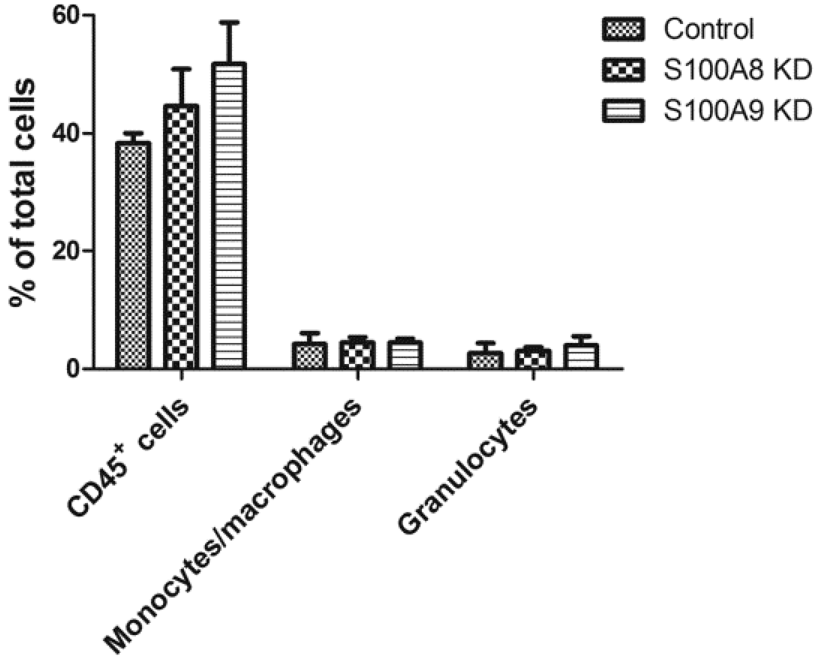

Supplementary Figure 8: Effects of S100A8 and S100A9 knockdown on myeloid cell recruitment in MC38 liver metastases. Single cell suspensions were prepared for FACS analysis from tumor bearing livers of control, S100A8 KD or S100A9 KD MC38-inoculated C57BL/6 mice. Percentage of $CD11b^+$ myeloid cells was analyzed by FACS, n=5 mice/group.
